# Monitoring environmental heat on urban green infrastructure in central Italy based on the florence case study

**DOI:** 10.1038/s41598-025-34090-4

**Published:** 2026-01-16

**Authors:** Arcangela Frascella, Giulia Guerri, Alfonso Crisci, Marco Dainelli, Cristina Gonnelli, Vergari Daniele, Marco Morabito, Sara Pignattelli

**Affiliations:** 1 National Research Council (CNR) Institute of Bioscience and Bioresources (IBBR), Via Madonna del Piano, 10, 50019 Sesto Fiorentino, Italy; 2 National Research Council (CNR) Institute of Bioeconomy (IBE), Via Madonna del Piano, 10, 50019 Sesto Fiorentino, Italy; 3 Department of Biology, University of Florence, Via Micheli 1, 50121 Florence, Italy; 4 Consorzio Di Bonifica 3 Medio Valdarno, Florence, Italy

**Keywords:** Heat stress, Chlorophyll-*a* fluorescence, Heat-exploratory model, Urban vegetation, Urban plant’s health, Ecology, Ecology, Environmental sciences, Physiology, Plant sciences

## Abstract

Urban green infrastructures (GI) are increasingly affected by environmental fluctuations which may impair photosynthetic performance and reduce ecosystem services. This study aimed to assess the health state of urban GI ecosystems in Florence, Italy, from May to October 2023, using different plant species (*Arundo donax*, *Laurus nobilis* and *Artemisia verlotiorum)* as bio-indicators monitored throughout their entire vegetative season. To this end, chlorophyll-a fluorescence (JIP-test), optical leaf traits, and meteorological indicators were integrated. Climate data showed frequent heat stress conditions, with 70–87% of summer days exceeding 30 °C and up to 45% surpassing 35 °C. *L. nobilis* exhibited the strongest heat sensitivity in July–August, showing a reduction in PI_ABS_, whereas *A. donax* and *A. verlotiorum* maintained FV/FM values within optimal ranges (0.75–0.85). Energy fluxes (ABS/RC, TRo/RC, DIo/RC) peaked in summer across species, indicating increased excitation pressure on PSII. Optical traits revealed species-specific strategies: *A. donax*, showed the highest chlorophyll content, particularly in October (1.1 ru, p < 0.001), while *A. verlotiorum* accumulated the most flavonols, with peak values in May and August (1.65 ru, p = 0.003), highlighting their photoprotective role under high irradiance and temperature. Linear models confirmed strong associations between heat predictors and variables such as PI_ABS _and FV/FM, supporting their value as indicators of thermal stress. Exploratory heat impact models revealed a clear relationship between environmental temperature and fluorescence-derived parameters, supporting their potential as sensitive indicators of urban thermal stress. These findings provide the basis for developing precision urban heat models for green infrastructures using chlorophyll fluorescence parameters combined with environment-based predictors.

**Supplementary Information:**

The online version contains supplementary material available at 10.1038/s41598-025-34090-4.

## Introduction

As sessile organisms, plants are exposed to various environmental fluctuations, making them ideal for continuously monitoring changing habitats^1^. The exposure to high temperatures combined with water shortage and anthropic activities pose significant threats to plant survival and growth^2,3^, lead to soil transformations and ecosystem degradation. Nowadays, cities frequently experience extended periods of low precipitation, resulting in increased water stress for urban vegetation. In addition, they are warmer than their surrounding regions, making heat stress a major limiting factor for urban Green Infrastructure (GI)^4^. GIs are the network of green spaces, water and other natural features within urban areas. A green infrastructure approach uses natural processes to deliver multiple ecosystem services, such as reducing the risk of flooding, cooling high urban temperatures and reducing air pollution level. It includes parks, playing fields, private gardens, allotments, green roofs and walls, and cemeteries. Plant ecophysiology gives us insights related to the plant capability to survive in surrounding environment, included strongly anthropized areas, thus managing to guarantee the fundamental ecosystem services that characterise green infrastructures^5^. The use of plants living in urban areas, such as bio-indicators, provide us useful information’s to understand the “urban” ecosystem status through the assessment of their eco-physiological state^6^. Plant’s physiological process, such as photosynthesis, is a potential fruitful way to evaluate the GI ecosystem services performance^7^. Photosynthesis is strictly regulated by environmental conditions such as temperature, water and light, which in turn undergo to the effects of climate change^8,9^. Moreover, photosynthetic activity can vary among plant species and genotypes. Plant species affects photosynthesis by evolving different carbon fixation strategies, such as the C3, C4, or CAM pathways, depending on their habitat. Furthermore, long-term exposure to specific environmental regimes led to additional adaptive modifications influencing photosynthetic efficiency^10^,^11^. Genetic variation can influence photosynthesis as well, leading to different phenotypic expressions arising from the same genotype^12^. A prolonged abiotic stress on the photosynthetic machinery impacts not only at plants level, but on bigger scale at ecosystem level^8^. During the photosynthetic process, light energy absorbed by photosynthetic pigments can be dissipated by both photosynthesis and non-photochemical processes, such as heat (lose by internal conversion) or fluorescence (light emission)^13^. The light energy that is dissipated by florescence is absorbed primarily by chlorophyll-*a* (Chl-*a*), a pigment mainly involved in the oxygenic photosynthesis. It has a role either as light harvester, than in both Photosystems (PSII and PSI) such as redox player in the charge separation carried out in the reaction centres (RCs)^14^. Chl-*a* fluorescence is a useful tool to assess the status and well-functioning of PSII, for this reason it is widely used for environmental and ecological studies^15^.

The GI under study, in Florence, represents a semi-natural area in the urban contest, traffic-free, serving as a space to recreational anthropic activities. Considering the rising temperatures caused by the climate change, already highlighted by international organizations such as the Intergovernmental Panel on Climate Change^16^ and the proximity of both the railway and highway, which may also contribute to the increased heat, there is a growing need to assess the “health status” of this area. Specifically, heat stress arises when high temperatures negatively affect a plant, and in temperate climates, it typically occurs within the temperature range of 35–40 °C^17^; however, variability among plant species must be also taken into account^18^.

The plant species chosen are those mostly growing in this area: *Arundo donax*, used to stabilize the riverbanks and avoid their slide down, furthermore it is uniformly distributed in this site; *Laurus nobilis* is one of the ornamental species mostly grown; and *Artemisia verlotiorum*, a species highly colonizing this area and uniformly distributed. On each plant species, during the growing season, in vivo analyses were carried out to evaluate their photosynthetic apparatus trough the JIP-test^19^. JIP-test give us a series of parameters, obtained from chlorophyll-*a* fluorescence analyses, describing the energy fluxes taking place inside and around the reaction centre (RC) of the photosystem II (PSII)^15^. Parameters analysed are those grouped in the efficiencies and quantum yield, which give us insight into the behaviour of PSII, highlighting the maximum quantum yield of primary PSII photochemistry (F_V_/F_M_), and the efficiency with which a PSII trapped electron is transferred: from Q_A_^-^ to PQ (ETo/TRo), to final PSI acceptors (REo/TRo), and efficiency with which an electron from PQH_2_ is transferred to final PSI acceptors (REo/ETo)^13^. Furthermore, to gain a better understanding about photosynthetic system response, parameters grouped in the specific energy fluxes per reaction centre (RC) were also analysed, such as absorption flux (ABS/RC), trapping flux (TRo/RC), electron transport (ETo/RC), reduction of the final electron acceptors (REo/RC) and the dissipation flux (DIo/RC). Finally, the performance index (PI_ABS_) was assessed to evaluate plant vitality, which is related to the primary photochemistry and electron transport flow due to its sensitivity to environmental stressor^15^. Additionally, chlorophylls (Chl), flavonols (Flv) and Nitrogen Balance index, that is the ratio between Chl and Flv, were also analysed in vivo^20^.

The purpose of this work is understanding the health state of urban GI ecosystem by using different plant species as bio-indicators, monitored throughout their entire vegetative season. This is achieved by the assessment of the photosynthetic apparatus and related metabolic compounds, in order to understand how various species growing in the same area respond to elevated summer temperatures. Results were than related to environmental temperatures of the area. Furthermore, for the first time, an exploratory model was developed by using photosynthetic parameters and metabolic compounds, while meteorological indexes, such as heat stress predictors were used to study their relationships.

## Results

### Climatic conditions during the monitoring period

The monitoring period (May–October 2023) was characterized by progressively warmer conditions from late spring through summer, accompanied by variable cloud cover. ERA5-Land monthly averaged data indicated that mean air temperatures ranged from 16 °C in May to 26 °C in July, with monthly minimum values between 8 °C (May) and 16.5 °C (July), and maximum temperatures between 25 °C (May) and 38 °C (August) (Table S1). Surface shortwave radiation peaked in July (243 W m⁻^2^), gradually decreasing to 105 W m⁻^2^ toward the end of the season.

Satellite-derived MODIS cloud cover data revealed a marked seasonal trend, with a progressive increase in clear-sky conditions from late spring to mid-summer. The number of clear-sky days (cloud cover < 10%) showed substantial seasonal variability, peaking in July (21 days, 68%) and remaining relatively high in August (19 days, 61%), while lower frequencies were recorded in June (11 days, 37%), October (26%), and May (29%), indicating reduced clear-sky conditions during the transitional months.

In addition, the occurrence of high temperatures was as follows: the percentage of days with maximum temperature > 30 °C was about 60% in summer (June–August), with the highest value (87%) in July. Days with extreme temperatures (> 35 °C) were less frequent: 3% in June, 32% in July, and 45% in August. In particular, during July and August, air temperatures frequently exceeded 30 °C (80% of the days) and 35 °C (40% of the days), combined with a high proportion of clear-sky days (approximately 65%) (Table S2).

Compared with the 1991–2020 climatic baseline, the 2023 growing season was consistently warmer than average across the May–October period. It was characterized by elevated air temperatures, with mean summer anomalies of approximately + 2 °C and a maximum anomaly of + 4 °C in October, which nonetheless featured generally mild temperatures, rarely exceeding 30 °C (only about 2% of the days). Minimum air temperatures remained above the long-term average throughout the monitoring period, with anomalies ranging from + 2 °C in June to + 5 °C in October. Maximum temperatures also exhibited marked deviations, particularly in August and October, with positive anomalies of + 4 °C and + 6 °C, respectively. Detailed information on clear-sky days, days with air temperatures exceeding 30 °C and 35 °C, and 2023 climatic data relative to the 1991–2020 baseline is provided in the Supplementary Materials (Tables S1 and S2).

### Chlorophyll-a fluorescence parameters

**Fig. 1:**
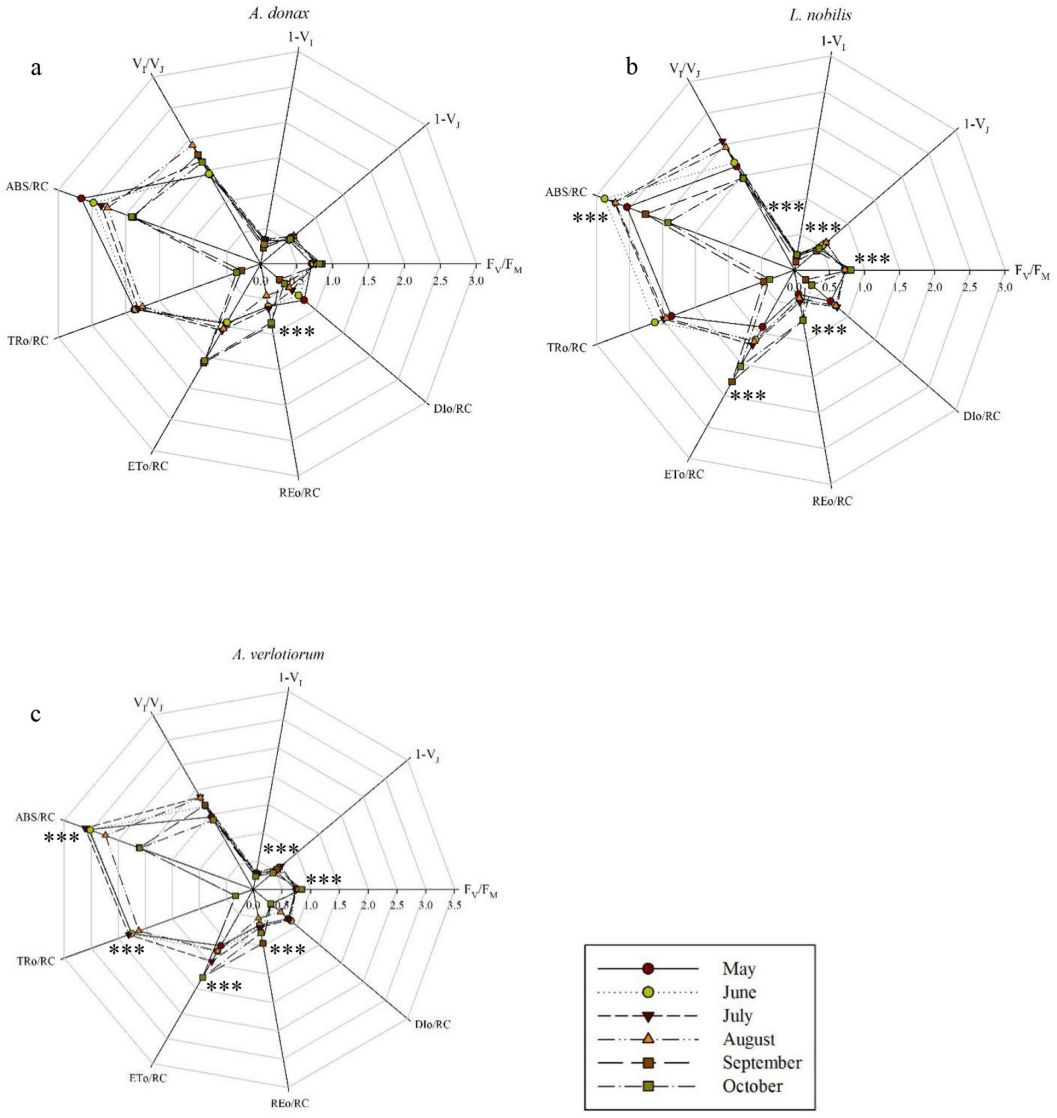
Spider plots of selected chlorophyll a fluorescence transients parameters measured in 72 young fully expanded leaves of (**a**) *A. donax*, (**c**) *A. verlotiorum* and 36 young fully expanded leaves of (**b**) *L. nobilis*. Each point represents the average value determined using one-way repeated measures ANOVA. Asteriks (*, **, ***) indicate significant differences between parameters during the monitoring season, according to Fisher LSD test at *p* < 0.05, *p* < 0.01 and *p* < 0.001, respectively.

The differences between specific energy fluxes per active reaction centre (ABS/RC, TRo/RC, ETo/RC, REo/RC, DIo/RC) and quantum yield of PSII (1-V_J_, 1-V_I_, V_I_/V_J_, F_V_/F_M_) in the different plant species are represented in Fig. 1, Fig. S7A and Table S3. From one- way repeated measures ANOVA, *Arundo donax*, resulted statistically significant only for REo/RC, with *p* < 0.001 (Fig. 1a, S7A and Table S3). This parameter showed the highest value in September and the lowest in August with 0.8648 and 0.4498, respectively.

**Fig. 2:**
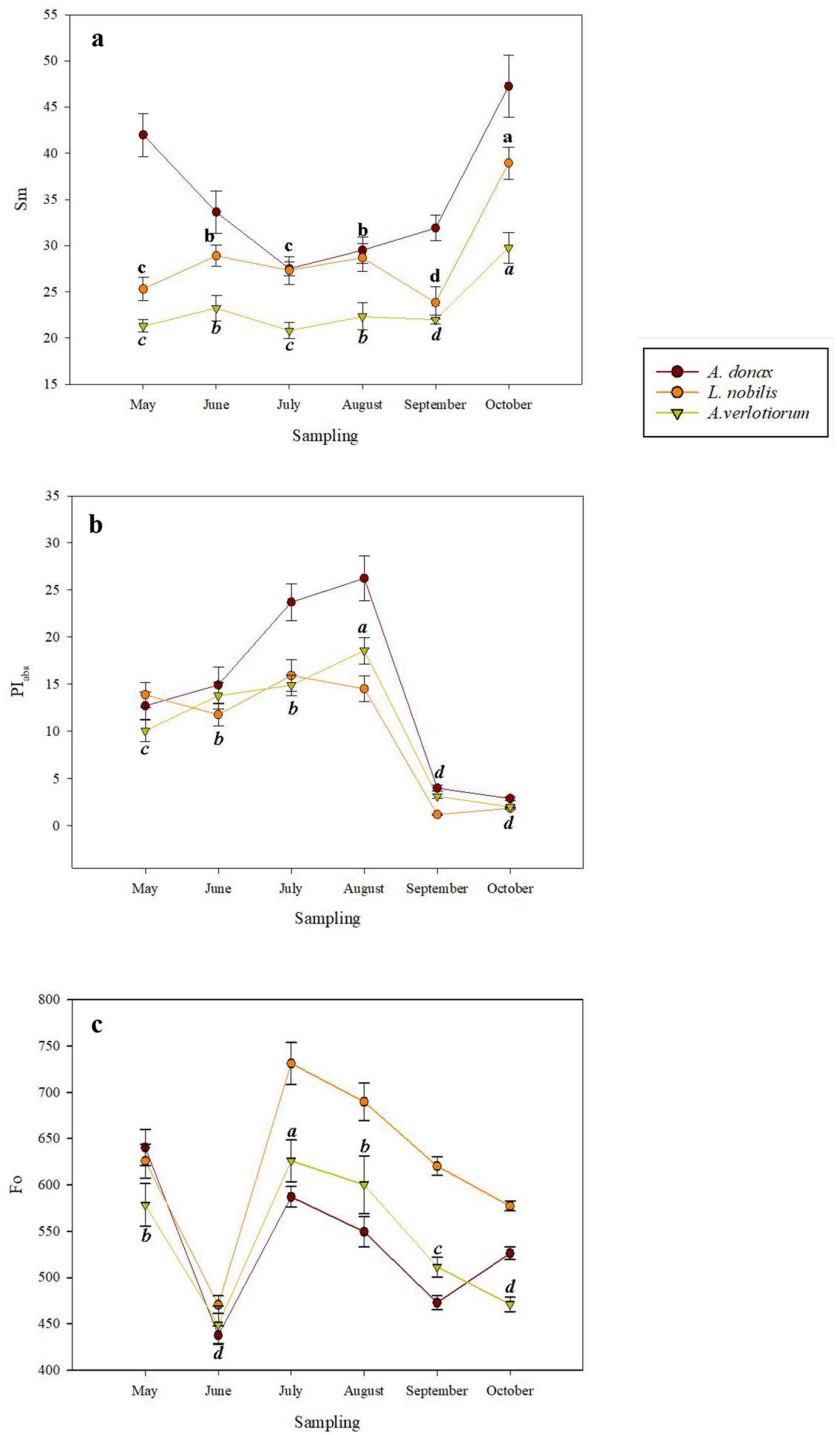
The trends, during the monitoring season of the (**a**) number of electron carriers per electron transport chain (Sm); (**b**) performance index, calculated on the energy absorption basis (PI_ABS_) and (**c**) minimal fluorescence (Fo), measured in 72 young fully expanded leaves of *A. donax*, *A. verlotiorum* and 36 young fully expanded leaves of *L*. *nobilis*. The graphs represent average values ± standard error at *p* < 0.05, as determined using one-way repeated measures ANOVA, followed by Fisher LSD test. Different letters indicate a significant difference along the monitoring season. In the graphs, lowercase letters referred to *L. nobilis*, italics lowercase letters referred to *A. verlotiorum*.

*L. nobilis* resulted statistically significant for the following parameters: F_V_/F_M_, 1-V_J_, 1-V_I_, ABS/RC, ETo/RC, REo/RC and Sm, with p < 0.001(Figs. 1b, 2a, S7C and Table S3). F_V_/F_M_ values ranged between 0.7 and 0.8 along the monitoring season, with the highest and lowest values of 0.8031 and 0.7159 recorded in October and July, respectively. 1-V_J_ measurements showed an increasing trend until July, with a value of 0.5926, month in which F_V_/F_M_ also showed the highest value. Conversely, the lowest value of 1-V_J_ was recorded in September with 0.4083. As expected, 1-V_I_ followed the same trend of 1-V_J_ but resulted twofold lower, with highest and lowest values observed in July and September with 0.2140 and 0.1165, respectively. As for parameters related with the specific energy fluxes per active PSII, ABS/RC displayed a decreasing pattern from June to October, with a maximum of 2.8764 and a minimum of 1.9181. ETo/RC exhibited higher values in September (1.7789) and lower in May (0.9094). REo/RC showed the same trend of ETo/RC as well, but with values 2 and threefold lower, recording the highest and lowest values in September and May, with 0.7055 and 0.3405 respectively. The Sm parameter (Fig. 2a) showed similar values from May to September, while the highest value was reached in October, 1.5-fold higher than in May.

**Fig. 3:**
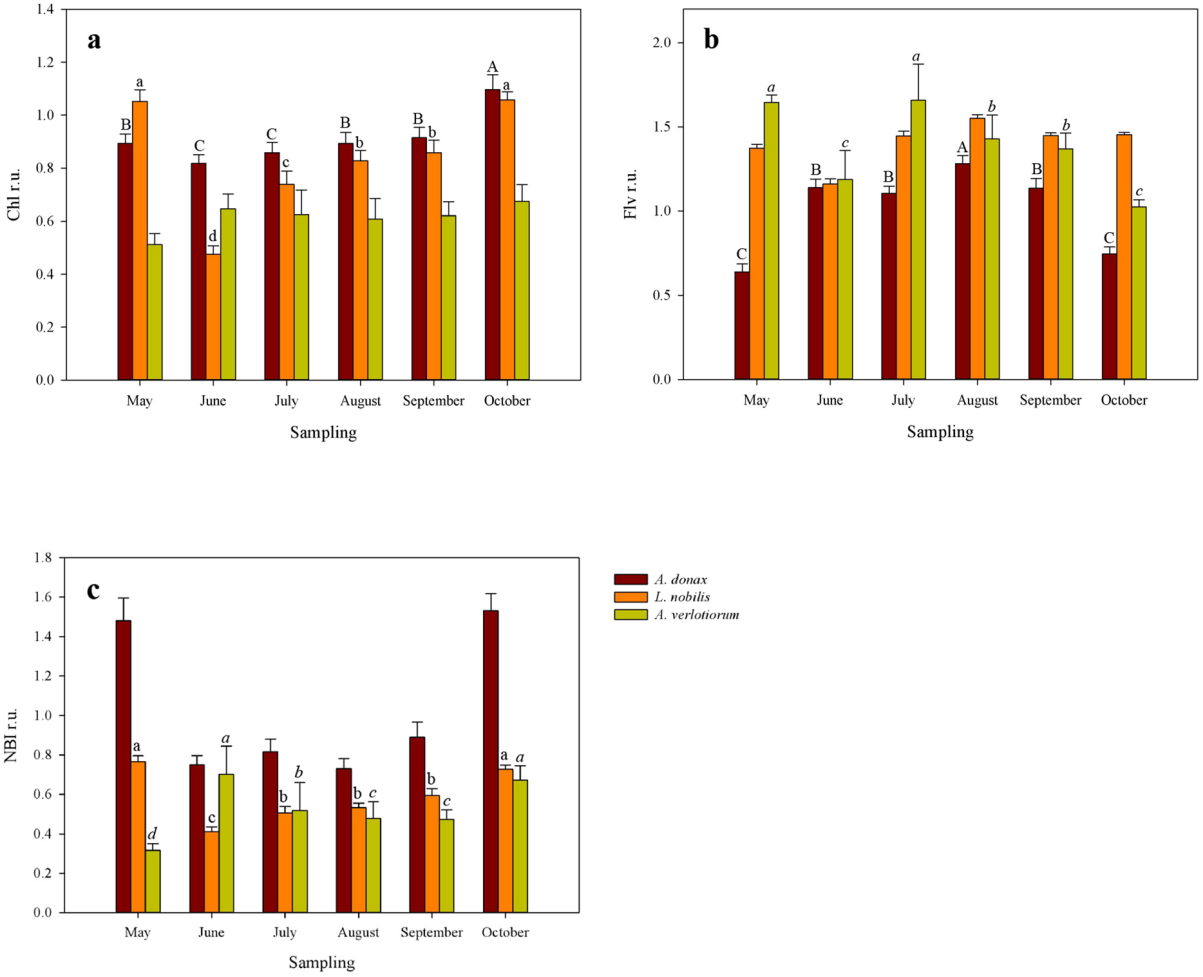
(**a**) Chl, (**b**) Flv and (**c**) NBI measured in 72 young fully expanded leaves of *A. donax, A. verlotiorum* and 36 young fully expanded leaves of *L. nobilis*, during the monitoring season. The histograms, related to each metabolite, represent average values ± standard error at *p* < 0.05, as determined using one-way repeated measures ANOVA, followed by Fisher LSD test. Different letters indicate significant differences between the species considered.

**Fig. 4:**
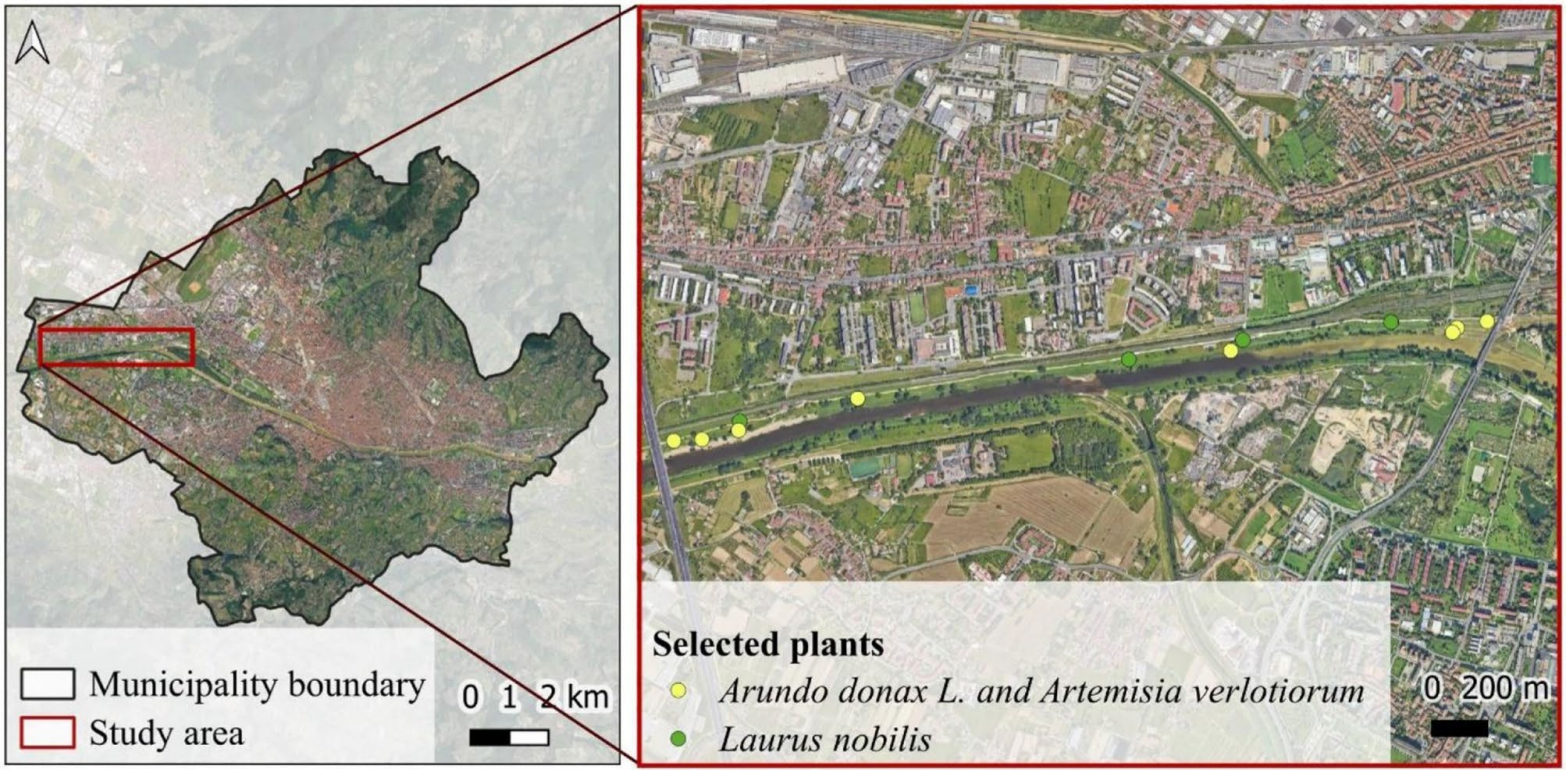
Map of the demonstrative area.

**Fig. 5:**
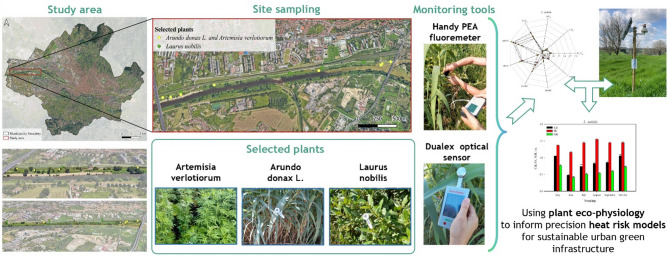
Schematic representation of the framework adopted in this study.

*A. Verlotiorum* resulted statistically significant for: F_V_/F_M_, 1-V_J_, ABS/RC, TRo/RC, ETo/RC, REo/RC (Fig. 1c), Sm, PI_ABS_ and Fo (Figs. 2 a, b, c, 3, 4c and 5c) with *p* < 0.001 (Table S3). F_V_/F_M_ displayed an increasing trend from May to October, showing values between 0.74 and 0.85 along the monitoring season. Interestingly, the highest F_V_/F_M_ values were recorded in *A. verlotiorum*. 1-V_J_, followed an opposite trend compared to F_V_/F_M_, it was lowest in October (0.4390) and twofold lower than F_V_/F_M_. On the other hand, the highest value was recorded in July (0.6272). Concerning the parameters related to the specific energy fluxes per active reaction centre, ABS/RC followed a mostly decreasing trend from May to October, although the highest value was reached in July with 3.1157. The same trend of the previous parameter was observed in TRo/RC. The values in autumnal season appeared noticeably lower than those recorded in summer, almost tenfold. For this parameter highest and lowest values are obtained for July and October with 2.3053 and 0.3292, respectively. ETo/RC registered highest value in October (1.7689) and the lowest in May (1.1337). A similar trend was observed for REo/RC, but with values about twofold lower, the lowest one recorded in August (0.5147) (Fig. S7B). The number of electron carriers per electron transport chain, Sm (Fig. 2a), appeared similar to those of *L. nobilis*, although with lower values. The highest value resulted in October (29.77) and the lowest in May, July and September (21.29, 20.80 and 21.98, respectively).

The performance index (Fig. 2b) showed an increasing trend until August, then decreasing up to October. *A*. *verlotiorum* was the only species statistically significant for Fo (Fig. 2c). Its season pattern revealed a predominantly decreasing trend, with the lowest value recorded in June (449).

### Chl and flavonols content and nitrogen balance index.

Trends and contents of chlorophylls (Chl), flavonols (Flv) and nitrogen balance index (NBI) varied between the species chosen. Chlorophylls resulted statistically significant for *A. donax* and *L. nobilis* with *p* < 0.001 (Fig. 3a and Table S4). Higher Chl contents are recorded for *A. donax*, followed by *L. nobilis* with increasing trends from June until to the end of the monitoring season. In October and May these species have showed higher values, with 1.1 and 1.05 of Chl contents for giant reed and laurel respectively; conversely, lower values are found in June with 0.81 and 0.47.

Species showing statistically significant differences for Flv are *A. donax* and *A. verlotiorum* with *p* < 0.001 and *p* = 0.003 respectively (Fig. 3b and Table S4). Lower Flv contents than all are recorded for *A. donax*. This species showed an increasing trend until August, months in which it showed higher content (1.28), to decrease until October; for giant reed the lowest flavonols value is recorded in May (0.63). In this latter month, *A. verlotiorum* recorded 2.5-fold higher Flv content than the previous species; furthermore, both in May and in August mugworth recorded higher Flv contents of the season (1.65). This species has shown a decreasing trend mostly, toward the end of the monitoring season, with lower values recorded in October (1.02).

NBI resulted statistically significant for *L. nobilis* and *A. verlotiorum* with *p* < 0.001 and *p* = 0.044 respectively (Fig. 3c and Table S4). These two species showed similar values for this parameter. *L. nobilis* recorded higher and lower contents of the monitoring season in May and June with 0.76 and 0.41 respectively; conversely *A. verlotiorum* recorded higher and lower values in June (0.70) and May (0.31).

### Estimation of thermal Impact on photosynthesis

Results from the linear models for each predictor related to photosynthetic activity of *L. nobilis, A. verlotiorum* and *A. donax* are presented in Tables 1, 2, and 3 respectively. The indices that showed the strongest association with the selected thermal predictors are primarily PI_ABS_ and F_V_/F_M_, with their higher predictability in terms of environmental thermal conditions. The determination coefficients (R^2^) of the models involving these two indices are 0.67 and 0.53 for *L. nobilis* (Table 1); 0.80 and 0.56 for *A. verlotiorum* (Table 2); and 0.56 and 0.41 for *A. donax* (Table 3). These parameters exhibited similar signs in the model coefficients, indicating a significant common response to thermal stress in the plant species chosen, both during the sampling day (lag0) and the previous one (lag7). The significance level of the models involving PI_ABS_ and F_V_/F_M_ is confirmed for each coefficient by p-values below 0.05. The other parameter considered, 1-V_J_, showed a significant association with thermal variables related to minimum temperatures (tmin_lag7) and temperatures on the sampling day (tmax_lag0) in *L. nobilis* (R^2^ = 0.45), and only with tmax_lag0 in *A. verlotiorum* (R^2^ = 0.68). The coefficient sign was negative for tmin_lag7, while it was always positive for tmax_lag0, as can be seen in the effect plots (Fig. S1 and S2). No linear association for this parameter was detected in *A. donax*.

**Table 1:** Summaries of linear models for* Laurus nobilis.*

Predictor	PI_ABS_	F_V_/F_M_	1-V_J_	NBI	Flv	Chl
*Intercept*	10.769 ***	0.747 ***	0.518 ***	0.572 ***	1.388 ***	0.799 ***
	(0.590)	(0.005)	(0.010)	(0.016)	(0.021)	(0.029)
*tmin_lag7*	-11.886 ***	0.065 ***	-0.116 ***	-0.035	0.018	-0.040
	(1.339)	(0.011)	(0.022)	(0.036)	(0.047)	(0.063)
*h_lag_o34*	-4.523 *	0.033 *	-0.021	0.119 *	0.042	0.178
	(1.884)	(0.015)	(0.031)	(0.053)	(0.069)	(0.092)
*tmax_lag0*	15.841 ***	-0.094 ***	0.155 ***	-0.095 *	-0.003	-0.130
	(1.537)	(0.012)	(0.025)	(0.042)	(0.054)	(0.073)
N	71	71	71	72	72	72
R^2^	0.670	0.537	0.454	0.458	0.030	0.311
F statistic	**45.255**	**25.864**	**18.588**	**19.156**	0.692	**10.230**
P value	**0.000**	**0.000**	**0.000**	**0.000**	0.560	**0.000**

All continuous predictors are mean-centered and scaled by 1 standard deviation. The outcome variable is in its original units. *** *p* < 0.001; ** *p* < 0.01; * *p* < 0.05. In parentheses, standard errors of the coefficients are reported.

**Table 2:** Summaries of linear models for* Artemisia verlotiorum.*

Predictor	PI_ABS_	F_V_/F_M_	1-V_J_	NBI	Flv	Chl
*Intercept*	12.013 ***	0.778 ***	0.552 ***	0.536 ***	1.356 ***	0.609 ***
	(0.497)	(0.005)	(0.007)	(0.061)	(0.073)	(0.033)
*tmin_lag7*	-10.022 ***	0.081 ***	-0.008	0.069	-0.158	0.056
	(1.106)	(0.012)	(0.015)	(0.135)	(0.163)	(0.074)
*h_lag_o34*	-9.678 ***	0.033	-0.028	0.152	0.045	0.056
	(1.602)	(0.018)	(0.022)	(0.196)	(0.237)	(0.107)
*tmax_lag0*	16.175 ***	-0.103 ***	0.075 ***	-0.167	0.294	-0.070
	(1.276)	(0.014)	(0.018)	(0.156)	(0.188)	(0.085)
N	54	54	54	54	54	54
R^2^	0.807	0.567	0.688	0.044	0.136	0.014
F statistic	**69.783**	**21.785**	**36.734**	0.761	2.632	0.228
P value	0.000	0.000	0.000	0.521	0.060	0.876

All continuous predictors are mean-centered and scaled by 1 standard deviation. The outcome variable is in its original units. *** *p* < 0.001; ** *p* < 0.01; * *p* < 0.05. In parentheses, standard errors of the coefficients are reported.

**Table 3:** Summaries of linear models for* Arundo donax.*

Predictor	PI_ABS_	F_V_/F_M_	1-V_J_	NBI	Flv	Chl
Intercept	15.496 ***	0.767 ***	0.547 ***	0.958 ***	1.064 ***	0.893 ***
	(0.767)	(0.005)	(0.009)	(0.039)	(0.026)	(0.020)
*tmin_lag7*	-13.256 ***	0.099 ***	0.006	-0.178 *	0.160 **	0.034
	(1.679)	(0.010)	(0.020)	(0.085)	(0.057)	(0.045)
*h_lag_o34*	-8.601 ***	0.047 **	0.020	0.383 **	-0.278 **	0.122
	(2.465)	(0.015)	(0.029)	(0.124)	(0.084)	(0.066)
*tmax_lag0*	21.243 ***	-0.108 ***	0.015	-0.214 *	0.090	-0.131 *
	(1.942)	(0.012)	(0.023)	(0.098)	(0.066)	(0.052)
N	143	143	143	143	143	143
R^2^	0.563	0.416	0.079	0.403	0.380	0.123
F statistic	**59.670**	**32.970**	**3.983**	**31.272**	**28.405**	**6.478**
P value	0.000	0.000	0.009	0.000	0.000	0.000

All continuous predictors are mean-centered and scaled by 1 standard deviation. The outcome variable is in its original units. *** *p* < 0.001; ** *p* < 0.01; * *p* < 0.05. In parentheses, standard errors of the coefficients are reported.

Regarding the variables related to NBI, Flv, and Chl, the linear models did not reveal any significant associations with thermal parameters in *A. verlotiorum*. For NBI, linear associations are evident with tmin_lag7 and tmax_lag0 in *L. nobilis* (R^2^ = 0.45), and with all parameters in *A. donax* (R^2^ = 0.40), though with slightly lower predictability, as indicated by the coefficient of determination. Only in *A. donax* were the variables tmin_lag7 and h_lag_o34 found to be associated with Flv levels, with a positive and negative sign, respectively. The Chl content only in *A. donax* was significantly and negatively associated with the parameter tmax_lag0. The linear contribution of each variable in the estimation of a specific predictor along with the corresponding margin of uncertainty, shown in the effect plots (Fig. S1 and S2 for *L. nob*ilis, Fig. S3 and S4 for *A. verlotiorum* and Fig. S5 and S6 for *A. donax*), was reduced in cases of significant association (i.e. PI_ABS_ and F_V_/F_M_).

## Discussion

The increase in temperature related to global warming is considered the main abiotic stressor, posing a significant threat to life on Earth. When temperatures exceed the average historical seasonal values, heat stress (HS) occurs^4^. Results obtained in this work by Satellite-derived MODIS, indicate that July and August represented periods of substantial heat stress, during which the sampled plants were frequently exposed to temperatures at or above their physiological tolerance limits. Specifically, the high frequency of days exceeding 30 °C (over 70% in July and August) suggests that plants were often subjected to conditions that could impair growth or trigger stress responses. The occurrence of extreme temperatures (> 35 °C) in July and August (32–45% of days) further highlights the potential risk for physiological damage, as reported in previous studies^17,20,21^. This combination suggests that the sampled plants were exposed to intense irradiance and thermal loads during the peak of the growing season. These conditions provide an essential context for interpreting the observed seasonal variations in photosynthetic performance and underscore the increased likelihood of heat stress in urban vegetation. Previous studies have shown species-specific temperature tolerances: for example, *Laurus nobilis* grows best in areas where annual daytime temperatures range from 17 °C to 25 °C, and can tolerate 8 °C to 30 °C^21^. In another case, *Artemisia sieberi alba* (a related species) showed marked physiological decline under combined heat and drought stress, demonstrating sensitivity to high-temperature conditions when air temperature exceeded 35 °C^22^. According to a recent study, *A. donax* can thrive in temperatures ranging from approximately 10 to 30 °C^23^. It is worth mentioning that the monitored riparian species, located along the Arno river in Florence, were not affected by water deficit. In this study, heat stress is therefore defined based on air temperature thresholds, occurring on days with air temperature above 30 °C and becoming more pronounced above 35 °C.

Compared with the last 30 years, the growing season under study was warmer. These deviations confirm that the sampling year experienced persistently above-normal thermal conditions, with the most pronounced anomalies occurring in mid- to late summer. This warmer-than-average context, particularly the high proportion of days with air temperatures above 30 °C and 35 °C in July and August, likely intensified the thermal stress experienced by the studied species. Although the study covers a single growing season, the pronounced heat-related stress responses observed are consistent with the increasing interannual climatic variability documented for Central Italy. Consequently, the physiological stress responses recorded in *L. nobili*s and, to a lesser extent, in *A*. *verlotiorum* and A. *donax*, occurred under a warmer-than-average climatic regime, in which prolonged heat and intense solar irradiance likely amplified the effects of summer stress on photosystem performance.

Photosynthesis represents the process most affected by high temperatures^24^. Heat stress is responsible for changes in the photosystem II (PSII) acceptors reduction–oxidation properties, and it can reduce the photosynthetic electron transport efficiency in both photosystems^15^. To evaluate plants’ health state and vitality by JIP test, the maximum quantum yield of photochemistry (F_V_/F_M_) is one of the parameters used; values between 0.75 and 0.85 indicate that the photosystem II is working well, conversely, lower values indicate that plants are under stress. When this condition is prolonged, PSII could be damaged irreversibly. The performance index (PI) is another parameter used as indicator of plant vitality and assesses the photosynthetic apparatus efficiency^25,26^. Previous studies, carried out on *Morinda citrifolia*, *Schoenoplectus tabernaemontani* and *Triticum aestivum*, showed that high temperature exposes plants to the risk of photo-damage resulting in a lower F_V_/F_M_ and PI, and increased fluorescence values when all PSII RCs are opened (Fo)^24,27,28^. This indicates that a disjunction of the PSII reaction centre from its pigment antennae occurred, causing a shutdown of energy transfer from the PSII traps. However, few studies have been conducted in urban environment by monitoring herbaceous and shrubby plants. In this study *L. nobilis* was the only species negatively affected by high temperatures, exhibiting F_V_/F_M_ values less than 0.75, in July and August. These reductions coincided with the highest air and dew point temperatures, that together with maximum solar radiation recorded in July and, more generally, from June to August, were coinciding with the months showing the greatest perturbations in plant photosynthetic efficiency. Furthermore, our results showed that to a decrease in F_V_/F_M_ corresponded to an increase in Fo, due to the higher temperature recorded during the monitoring season, as also reported in previous findings on *Triticum aestivum*^28^. Thus, the reduction observed in the maximum quantum yield, in the warmer months, can be caused by a decreased PSII photochemical efficiency due to higher temperatures, suggesting the onset of heat stress. Higher temperatures inhibit the redox reaction following Q_A_ and consequently delaying the electron transport between Q_A_^-^ and Q_B_^26^. The other two species chosen, *A. donax* and *A. verlotiorum*, have shown optimal values in July and June respectively, until the end of the monitoring season, evidencing that for these species, high temperatures do not represent a limit for their growth and development, since they are capable of acclimating to the urban heat stress^27^. The energy flux ratios are parameters describing the energy absorbed (ABS), trapped (TR) transported (ET) and dissipated (DI) as heat^29^. In previous works, it was already evidenced that these parameters change as a function of the stress provided ^26–32^. In our results, for all plant species considered, we detected a decreasing trend, towards the autumnal months, for ABS/RC, TRo/RC, and DIo/RC, while ETo/RC followed a totally opposite pattern. ABS/RC is the ratio between the total amount of photons absorbed by chlorophylls for all RCs and the total number of active RCs. It is affected by active/inactive RCs ratio where the increase in inactive (non-reducing Q_A_) RCs correspond to an increase in ABS/RC ratio^32^. This was in line with our results obtained during summer months, indicating an inhibition of electron transport from Q_A_^-^ to Q_B_, with a consequent conversion of RCs in ‘silent’ RCs^30^. Conversely, lower ABS/RC values obtained in autumn indicate a switch in favour of active (reduced Q_A_) RCs occurred. TRo/RC represents the maximum speed in which an exciton is entrapped by the RC, arising in a decreased Q_A_. An increase in TRo/RC ratio suggests an over-reduction of all Q_A_, as noticed in summer months, assuming that slower reoxidation of Q_A_ depends on the high temperature recorded in this season. The higher values found in the warmer months for both ABS/RC and TRo/RC suggest that some RCs were inactivate due to a prolonged inhibition of the oxygen-evolving complex (OEC) which can bring to OEC impairment^30^. However, a decrease of TRo/RC toward the autumnal months indicate that non-reducing Q_A_ takes place in favour of reduced ones, and OEC activation occurred back^27^. The ETo/RC is related to the Q_A_ re-oxidation through the electron transport in an active RC. As it is informative only for active RCs, the increase of ETo/RC during autumn indicates an increase in inactive centres and Q_A_ inability to transfer electrons efficiently to Q_B_, so indicating a decrease in the electron’s efficiency^29,32^. DIo/RC is the ratio between the total dissipation of untrapped excitation energy becoming from all RCs and the number of active RCs. In summer, higher DIo/RC values with respect to autumn values were observed, corresponding to a high dissipation ratio by active RCs. However, high DIo/RC also occurs when there is an increase of inactive RCs, as is related to the inability to trap photons^32^. Furthermore, DIo/RC and ETo/RC followed opposite trends: higher values of DIo/RC correspond to lower ETo/RC values, indicating that energy in excess is dissipated and highlighting a possible strategy of the plant to avoid photodamage to the photosynthetic machinery^30^.

The increasing PI_ABS_ values highlighted for *A. donax* and *A. verlotiorum* until August, indicate a raising of the metabolic activity due to the exit from winter dormancy^25^. Conversely, the decreasing trend recorded from August to October, could suggest a progressive slowdown of the metabolic activity, followed by the winter dormancy. *L. nobilis* showed the lowest values among the plant species considered, in a decreasing trend from July to October, thus resulting one month in advance if compared with the previous two species. Considering the decreasing trend such as the beginning of the progressive slowdown of the metabolic activity, is evident that for *L. nobilis* the decrease in metabolic activity occurred from July, month in which the highest temperatures were recorded; suggesting that for this species the decreasing pattern can be associated to heat stress occurred from July.

*A. Donax* and *A. verlotiorum* recorded the higher and lower chlorophylls (Chl) content, respectively, during the monitoring season. These species have shown also, the lower and higher flavonols (Flv) content respectively. Flv are a class of flavonoids, they work as antioxidants to counteract a huge range of stressors, between them, it is well known that high temperatures and high-light stress trigger the production of these secondary metabolites^33,34^. Higher Flv amounts, evidenced in July and August for *A. verlotiorum* and *L. nobilis*, confirmed their photoprotective role against high temperatures and solar radiations at leaf level^20^. Flv exhibited an opposite trend if compared with Nitrogen Balance Index (NBI); such as all polyphenols, Flv are inversely related with nitrogen leaf content because production of these carbon-based metabolites enhances when N is low^35^, in fact our results shown that at higher NBI levels correspond lower Flv levels, and vice-versa.

The exploratory models previously implemented have shown a strong linear association between photosynthetic efficiency, performance index and thermal predictors obtained from meteorological time series of air temperatures. This was observed in each species considered. However, the other predictor (1-V_J_) highlighted a weaker species dependent association. The effect of the parameter related to the minimum temperature (t_min_lag7) is species dependent, in fact *L. nobilis* resulted twofold higher than the extreme temperature exposure (h_lag_o34); confirming its higher sensitivity to the heat stress if compared with *A. donax* and *A. verlotiorum*, as previously noticed. The high coefficient ratio among predictors works such an alert of species heat sensitivity. In *A. donax* and *L. nobilis*, 1-V_J_ is more related to t_min_lag7 predictor; confirmed by the significance degree of the coefficients in the respective linear models (Tables 1 and 3). The associations related with Flv, Chl and NBI were week, thus not providing a coherent frame able to be explained, such as those mentioned above. Overall, the results outline a picture of generalized thermal stress throughout the monitoring period in an urban setting affected by elevated and rising temperatures, highlighting the persistence of heat conditions as a significant factor that should not be overlooked.

This investigation revealed that urban heat stress on GI at mid-latitudes, linked to the increasing frequency of high temperatures caused by climate change, impacts the photosynthetic apparatus by amplifying absorbed, trapped, and dissipated energy fluxes across all the species studied, particularly during the warmer months. Thus, the increase in the above-mentioned parameters can be due both to the high temperatures recorded, but also to the plant life cycle, confirming that the summer season represents a critical period for urban GI. These findings are supported by the linear exploratory models, developed for the first time and fed by measured meteorological indexes as thermal predictors obtained from time series of air temperatures; evidencing a strong association between the parameters considered. Furthermore, this work has laid the foundation for developing additional more comprehensive models by incorporating chlorophyll fluorescence parameters alongside other weather-based predictors. The comprehensive study design included the measurement of optical leaf traits, with flavonols emerging as key compounds in the investigated species. Their values increase particularly during the warmer months of the season, further confirming their protective role against high solar irradiance with elevated air temperatures. These conditions are typical of the summer months (June–August) at the sampling site, which are generally characterized by clear skies and the high incoming solar radiation typically observed in Central Italy. Finally, the results of this work suggest that monitoring urban heat stress in plants could be made feasible by developing customized models using data flows provided from sensor networks calibrated with fluorescence data. Future research could benefit from incorporating complementary parameters such as the Normalized Difference Vegetation Index (NDVI) and canopy temperature (T_canopy), which are widely used to assess vegetation stress and can help bridge the gap between leaf-scale measurements and landscape-scale patterns. In addition, imaging approaches described by Jones and Vaughan (2010)^36^ offer promising tools to enhance spatial resolution and diagnostic capability by providing continuous, non-destructive assessments over larger areas. Furthermore, due to the lack of some results such as plant and soil water status, in addition to the above-mentioned parameters, future monitoring research could be addresses on the soil and plants water status measurements, proximal thermography, leaf gas exchange and soil enzymatic activity, to have a complete and more deep knowledge of the status of the area under study.

## Material and methods

### Description of site sampling

The demonstrative area is located in Florence, along a semi-natural area on the right bank of the river Arno (central position of left end: 43° 47′ 15.23’' N 11° 09′ 33.49’' E; central position of right end: 43° 47′ 25.37’' N, 11° 11′ 47.55’' E) (Fig. 4). The study area is East–West oriented and extends along the right bank of the Arno River, a semi-natural zone that serves as a levee for the largest watercourse in Florence. The soil taxonomy of the area is fluventic haplustepts, coarse-loamy over sandy or sandy-skeletal, mixed, mesic (11° ed. 2010). It is a very deep soil with an Ap-Bw-2C-3BC profile, non-gravelly, with a texture ranging from sandy loam to silty clay loam in depth; calcareous, from slightly alkaline to moderately alkaline in the deeper layers, and well drained. In terms of hydraulic characteristics, it results with a moderate to high available water-holding capacity for plants, a saturated hydraulic conductivity ranging from moderately high to high (at least down to 90 cm depth), with predominantly vertical water flow, and the absence of a groundwater table within the first 150 cm. It also shows a very high rainfall infiltration capacity^37^**.** The demonstrative area stretches approximately 4 km in length (covering 19 hectares in surface area), has predominantly flat morphology (with an average elevation of 38 m a.s.l.), and is surrounded by numerous significant urban infrastructures: to the East and West by road and highway networks, to the North by the railway, and to the South by the Arno River. To assess the plant health along the area, the most frequently observed plant species were selected (*Laurus nobilis*, *Artemisia verlotiorum*, *Arundo donax L*.).

### Climatic data acquisition and processing

To provide a detailed climatological context for the interpretation of plant photosynthetic performance, ground-based meteorological observations with reanalysis climatic data and satellite products were integrated. Ground-based measurements were obtained from a weather station located near the study area, within 2 km distance from the sampling site. Based on this weather station data, days exceeding maximum air temperature values of 30 °C and 35 °C were extracted. In this study, heat stress is specifically associated with temperature thresholds, defined as days with maximum air temperatures exceeding 30 °C (days during which stress conditions may potentially occur), and is more likely on days with temperatures above 35 °C, following the previously mentioned studies^17,22,23^.

For the broader climatological characterization of the study area, the ERA5-Land monthly averaged dataset^38^ was used. ERA5-Land provides consistent, long-term climate reanalysis data at approximately 9 km spatial resolution and hourly temporal resolution, corrected for topography and surface characteristics. From this dataset, we extracted monthly mean, minimum, and maximum air temperatures (°C), surface shortwave radiation (W m⁻^2^), and soil temperature in the upper soil layers (up to 289 cm, as available in the reanalysis outputs).

The analysis covered the period from May to October 2023, corresponding to the growing season monitored for the selected species. As a reference, we used the 30-year climatological baseline (1991–2020) from the same ERA5-Land dataset. Monthly temperature anomalies (mean, minimum, and maximum air temperatures) for 2023 were calculated relative to the 1991–2020 climatological mean, using the expression ΔT = (T₂₀₂₃ – T₁₉₉₁-₂₀₂₀). All data were spatially averaged over the ~ 9 km grid cell encompassing the study site (43°47′ N, 11°10′ E).

To assess the potential influence of solar irradiance, we also analyzed satellite observations from the Moderate Resolution Imaging Spectroradiometer (MODIS) platform. Specifically, the number of clear-sky days per month (i.e., days with total cloud cover < 10%) was derived from MODIS Level-3 daily products for the same period and geographic area (Florence, Italy). These data provide a reliable indicator of exposure to direct solar radiation and complement the ground-based temperature information.

The combined use of ERA5-Land reanalysis and MODIS satellite datasets ensured a comprehensive characterization of local climatic conditions potentially influencing photosynthetic performance in urban vegetation.

### Optical and eco-physiological measures

In vivo measurements were taken on three leaves of a representative pool of each plant species once per month (24 plants of *A. verlotiorum*, *A. donax* and 12 plants of *L. nobilis*), from May to October during the morning between 9:00 and 12:00 am^39,40^. Plants’ eco-physiological state was verified, measuring the chlorophyll α fluorescence and, in real time, the chlorophyll content, flavonoids and nitrogen balance index during the seasonal and vegetative cycles of plants. Prior to the beginning of the monitoring season, the last internode was marked on each selected *A. donax* and *A. verlotiorum*. For each *L. nobilis*, a lateral branch was selected, and its terminal (younger) portion was marked. Subsequently, three of the youngest leaves were selected each month for in-vivo analyses. Chlorophyll α fluorescence measurements were carried out by means of a Handy PEA (Plants Efficiency Analyser, Hansatech Instruments, King’s Lynn, Norfolk, UK) fluorometer. Leaves were dark-adapted for 20 min before recording the fluorescence emission using leaf clips, then were illuminated with 660 nm light of 3500 μmol m^−2^ s^−1^ for 1 s. The measured fast chlorophyll fluorescence induction curves (Fo to Fm) were analyzed by the JIP test, based on the theory of energy fluxes in the photosynthetic apparatus. Fm is referred to the maximal fluorescence intensity when all RCs are closed, while Fo represents the minimum fluorescence intensity when all RCs are opened^32^. Data obtained from Handy PEA was extracted using the PEA Plus software application (v. 1.13). The list of parameters is reported in supplementary data (Table S5).

Chlorophyll (Chl), flavonols (Flv) and the derived nitrogen balance index (NBI) were monitored by a portable Dualex® instrument (Multi-Pigment-Meter MPM-100, Paris, France). The flavonols of leaf epidermis and chlorophyll contents are provided by the ratio of fluorescence to measure flavonol content (ratios: F660nm/F325nm) and leaf transmission in the near and far infra-red to determine the chlorophyll content (T850/T720). NBI is calculated as the ratio between chlorophyll and flavonol^41^. All these indices are expressed in relative unit (r.u.).

### Estimation of thermal impact on photosynthesis

To assess the level of thermal stress experienced by the sampled plants, a series of linear models were developed using indices based on chlorophyll fluorescence measurements and other parameters related to pigment content. Specifically, the following indices were used as variables: F_V_/F_M_, PI_ABS_, 1-V_J_ (Tab S5), NBI, Flv and Chl.

Based on the sampling dates, two temporal windows were defined: the first (lag7) corresponds to the seven days preceding the sampling date, while the second (lag0) covers the first 12 h of the sampling day.

The following thermal predictors were derived from the hourly data of the “Firenze Università” station:

- tmin_lag7: The absolute minimum air temperature over the 7-day period (lag7)
- h_lag_o34: The number of hours with air temperatures above 34 °C during the 7-day window
- tmax_lag0: The maximum air temperature measured during the first 12 h of the sampling day (lag0)

The first two predictors account for thermal stress from potential heat exposure, affecting both minimum and maximum temperatures in the days leading up to the measurement, while the third represents the thermal stress experienced on the day of sampling.

To avoid introducing collinearity into the linear regression models, no additional thermal predictors were included. In total, six models were developed for the three plant species under study. Data values used to model are summarized in Table S6.

### Statistical analysis

Descriptive statistics (means, standard errors) were performed for all measured parameters using SigmaPlot 12.5 (SPSS Inc., Chicago, IL) scientific data analysis and graphing software. All data were assessed for normality by means of Kolmogorov-Smirnoff statistic (p > 0.01); then One-way repeated measures ANOVA (analysis of variance) was applied to verify differences between time, parameters analysed by non-destructive analyses and their interaction. The statistical significance was set at p < 0.05 according to Fisher LSD test.

The statistical relative to linear modeling was conducted by using the lm function in the R environment^42^. The following R packages were used to manage and visualize the model outcomes:

- sjPlot: for plotting model results^43^
- effects: for generating effect plots^44^

The model results are summarized in three comprehensive tables, one for each species, providing the following information:

- The values and significance levels of student’s t-tests for each individual predictor
- The determination coefficient (R^2^) of the model, indicating the amount of variance explained by the model
- The significance level of Fisher’s Test (F statistic)

Finally, the effects plot for each predictor and species were presented. Effect plots display the mean response of the predictor variable across its data range. The sign and magnitude of the effect indicate the direction and strength of the relationship between the predictor and the photosynthetic variables, offering an effective visualization of the information provided by the fitted models.

Figure 5 illustrates the framework adopted in this study.

## Supplementary Information

Supplementary Information.

## Data Availability

The datasets generated and/or analysed during the current study can be obtained from the corresponding author upon reasonable request.
