# Supplementary material for: Monitoring environmental heat on urban green infrastructure in central Italy based on the florence case study
Source: Sci Rep. 2026 Jan 16;16:4041. doi: 10.1038/s41598-025-34090-4 (PMC12855989; doi:10.1038/s41598-025-34090-4)
Supplement: Supplementary file 1 — Supplementary Information. [file 41598_2025_34090_MOESM1_ESM.docx]

Figure S1. Effect plots of photosynthetic predictors for *Laurus nobilis*


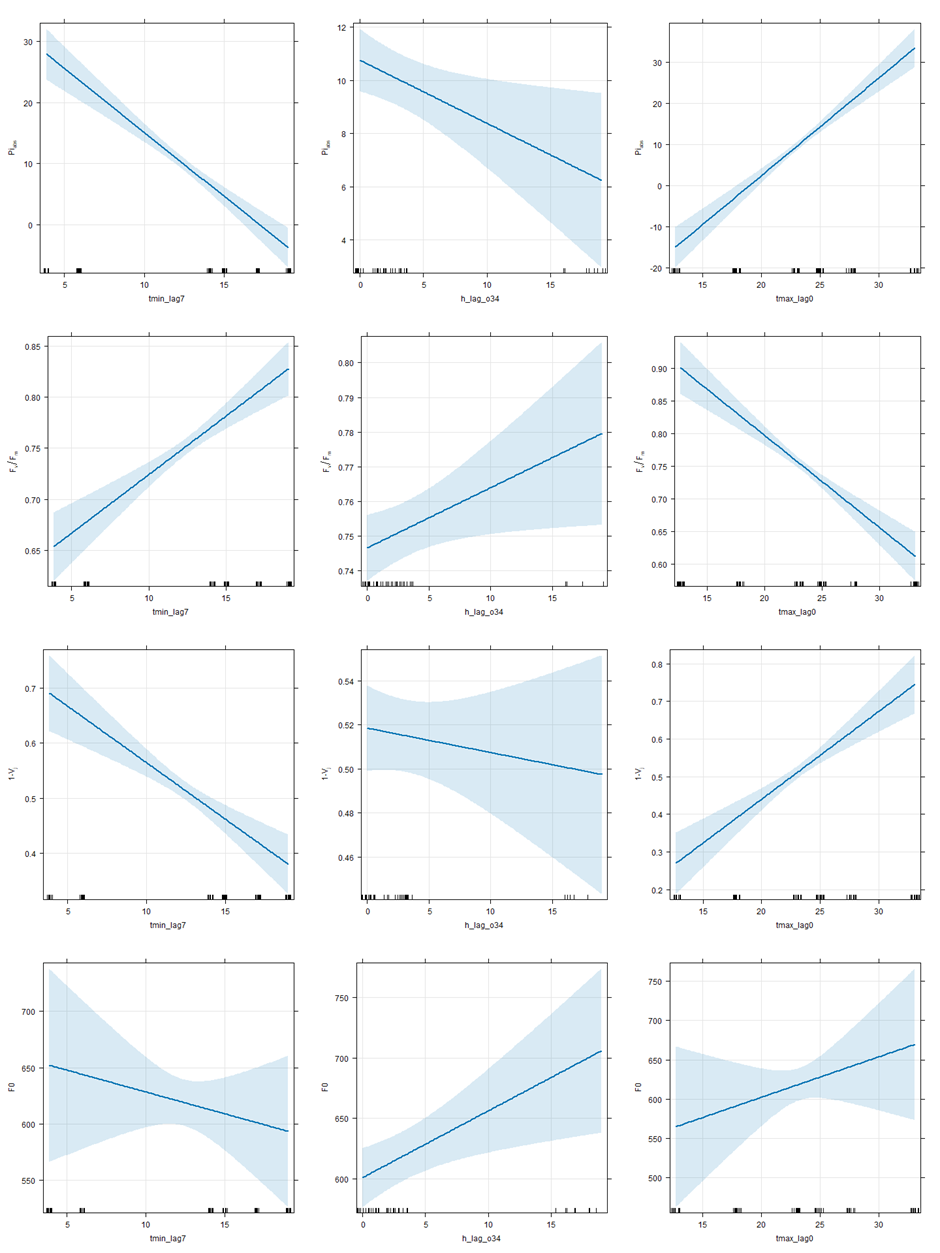


Figure S2. Effect plots of pigment-related predictors for *Laurus nobilis*


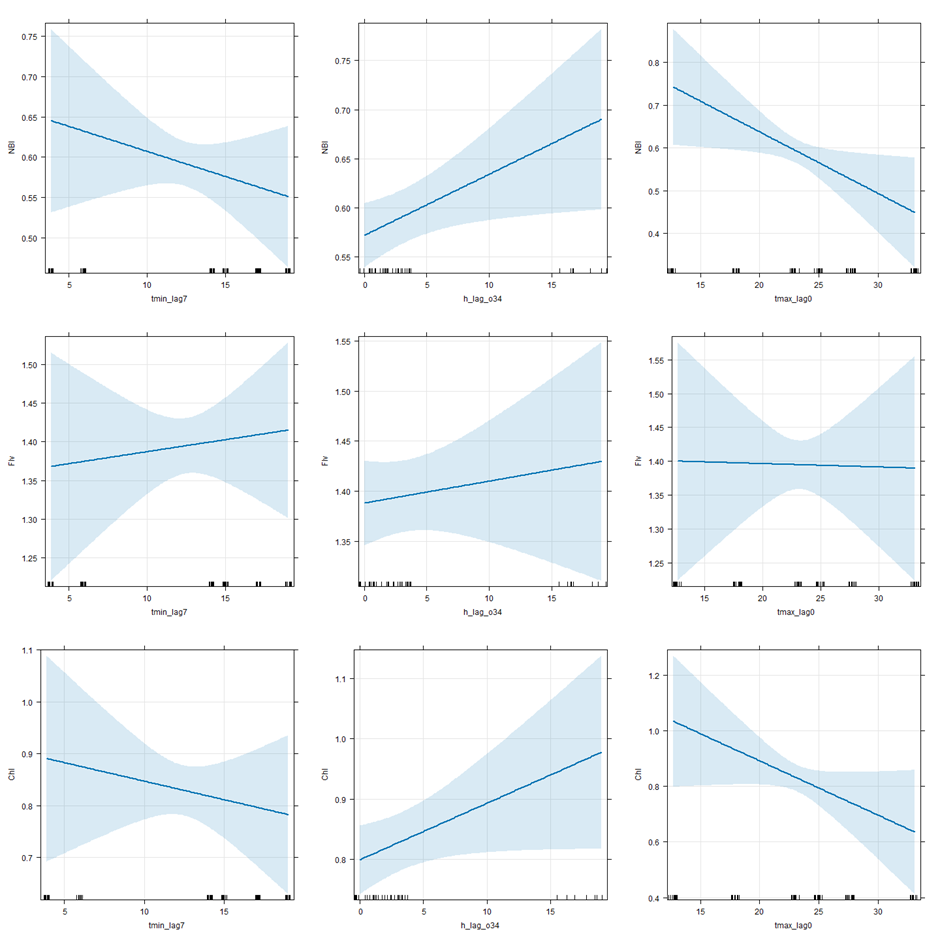


Figure S3. Effect plots of photosynthetic predictors for *Artemisia verlotiroum*


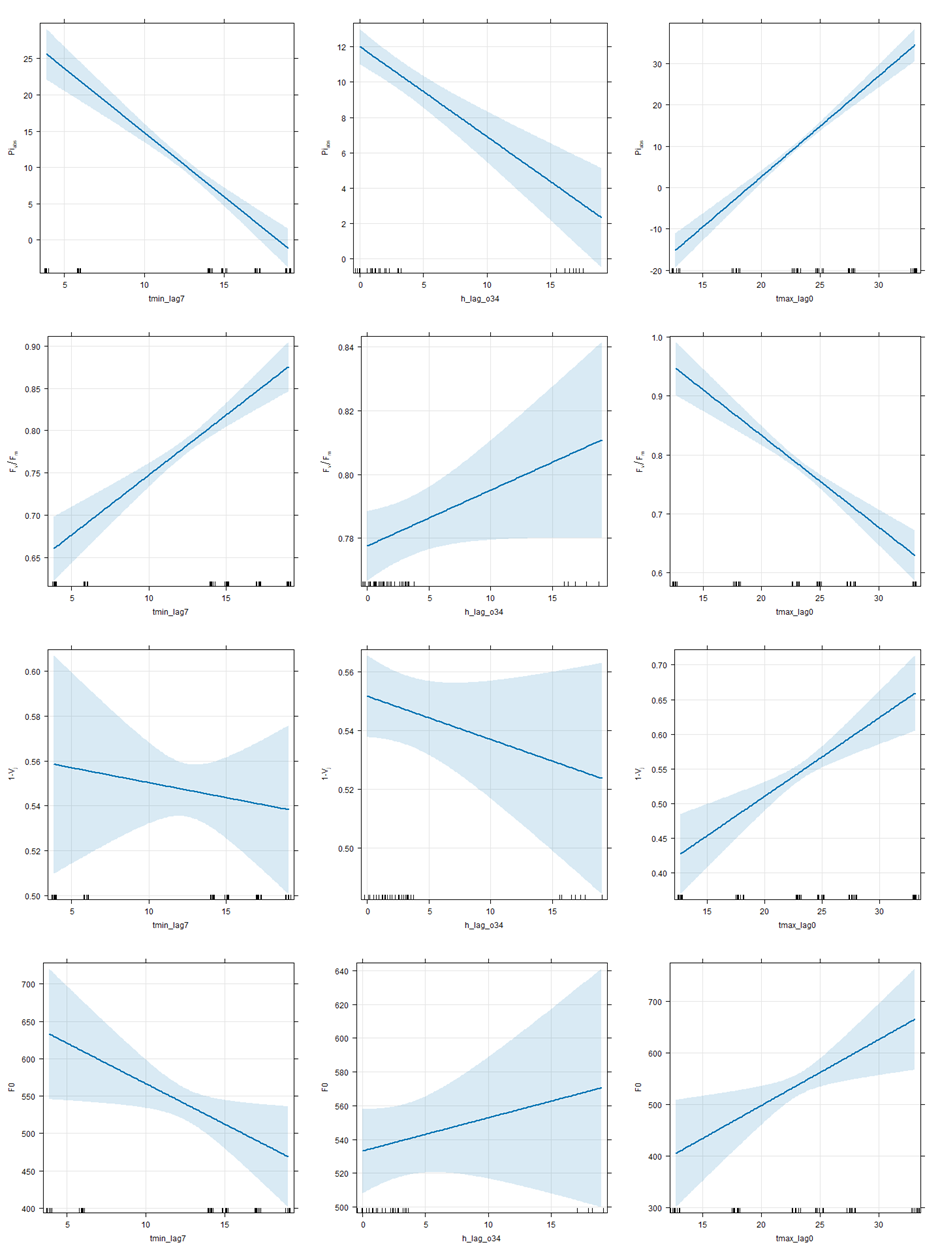


Figure S4. Effect plots of pigment-related predictors for *Artemisia verlotiorum*


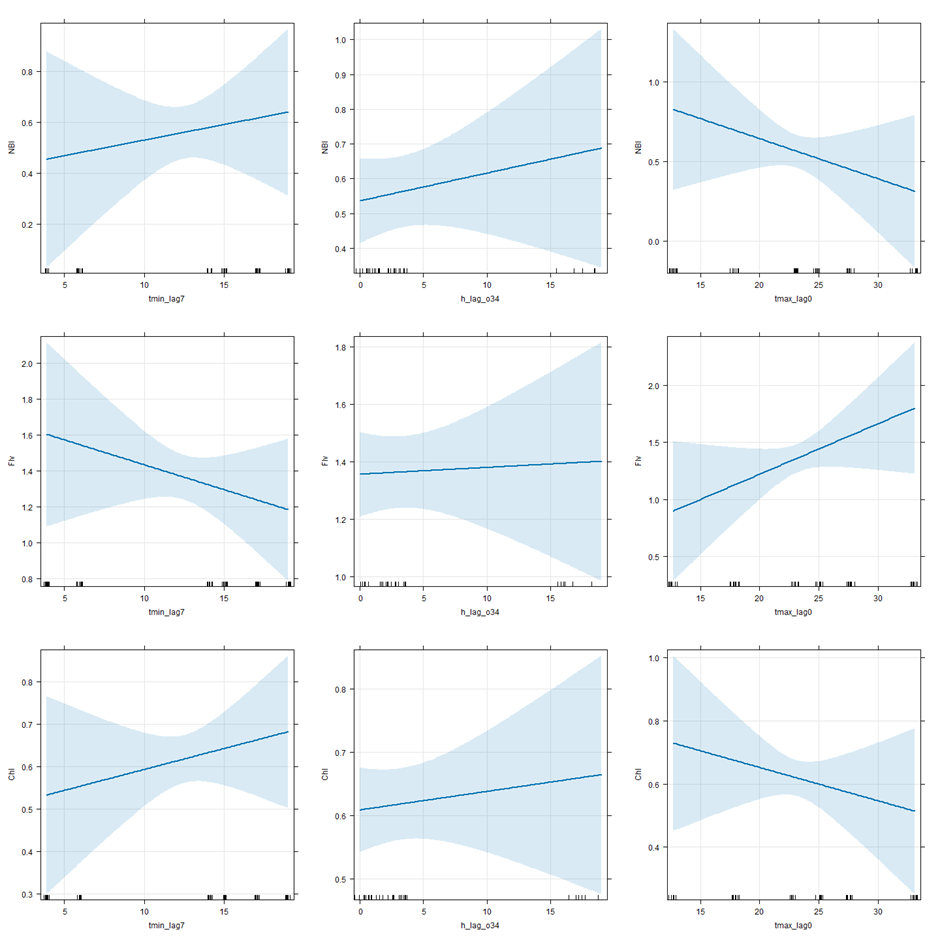


Figure S5. Effect plots of photosynthetic predictors for *Arundo donax*


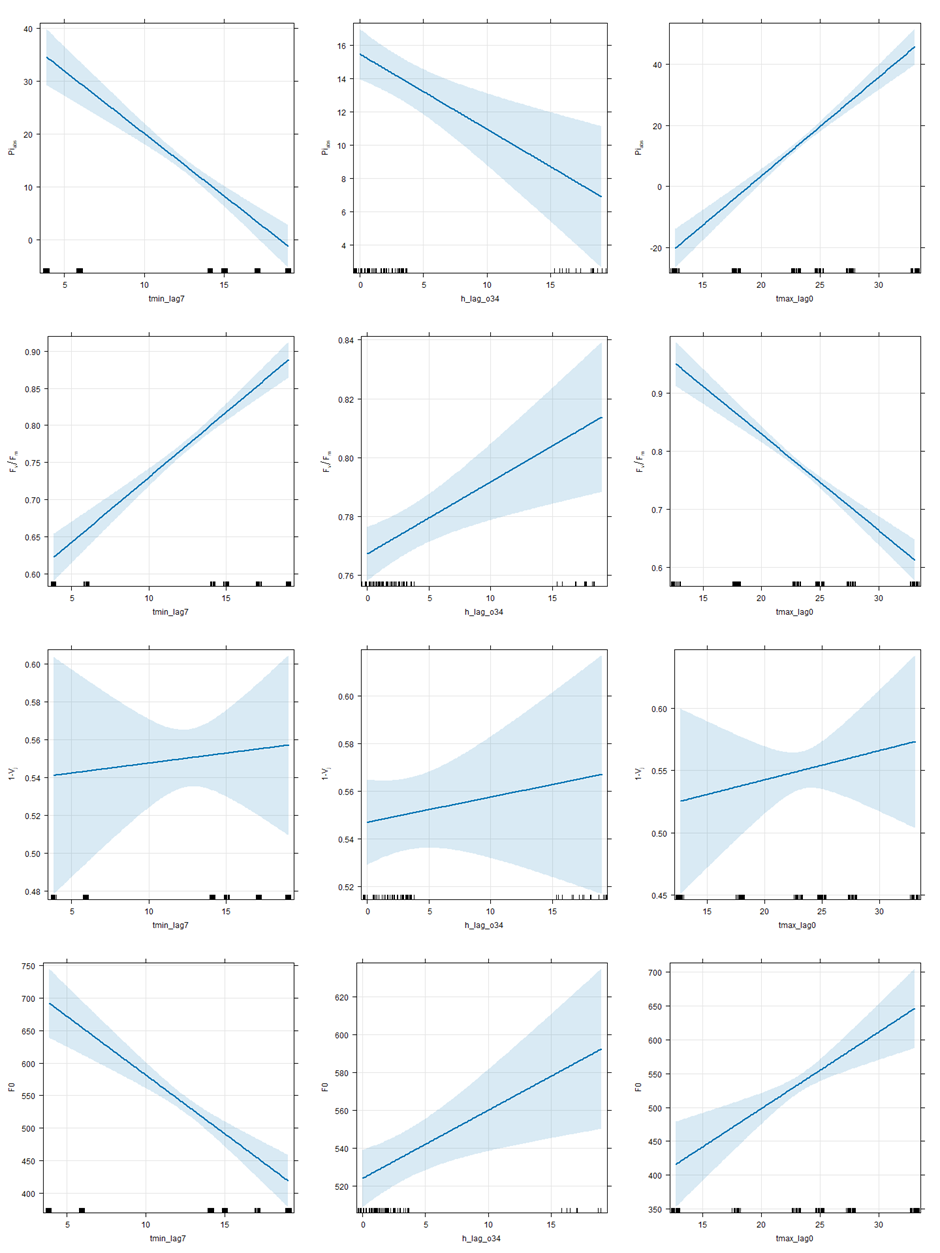


Figure S6. Effect plots of pigment-related predictors for *Arundo donax*


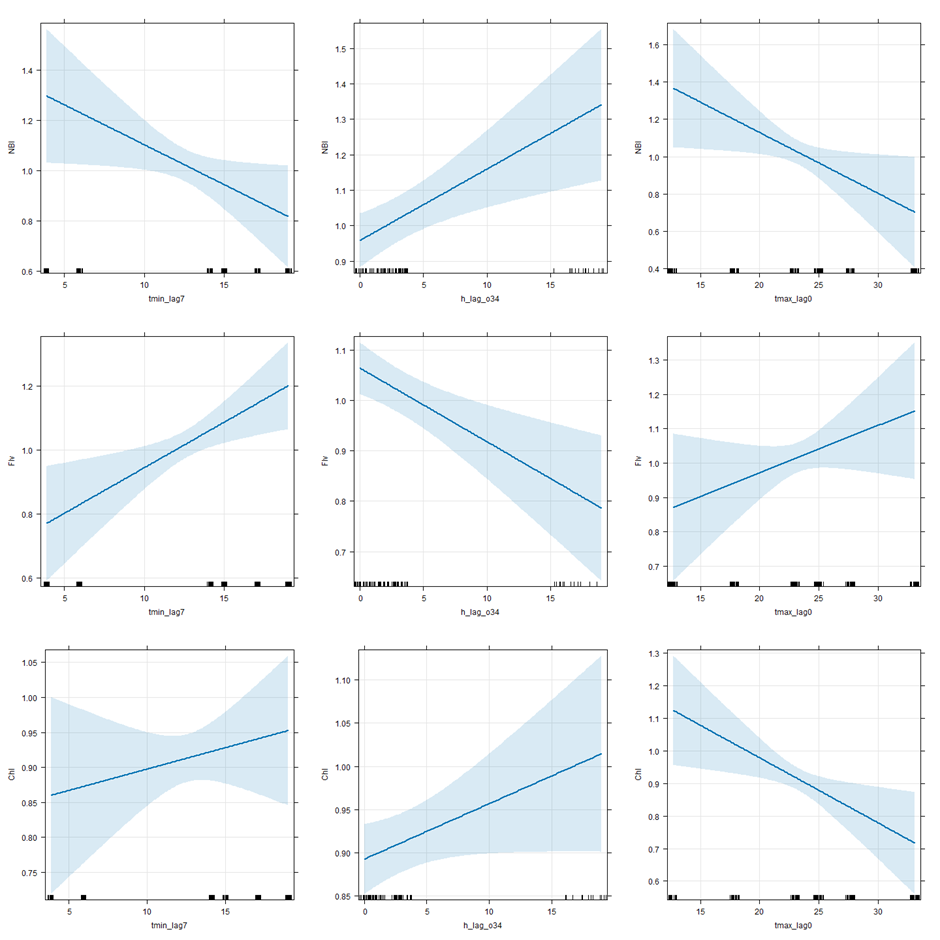


Figure S7 seasonal trends of each Chl-a parameters showed in the spider plot for A) *A. donax,* B*) A. vulgaris* and C*) L. nobilis*


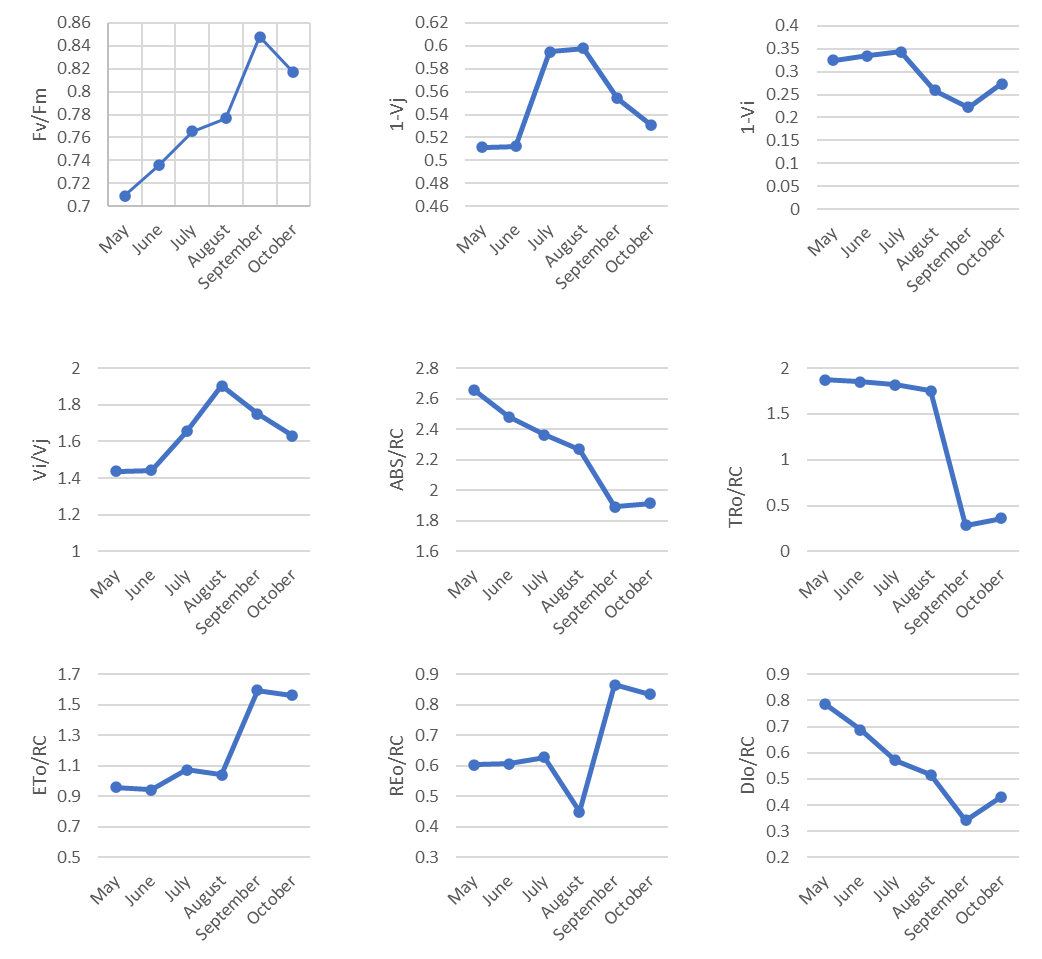


A


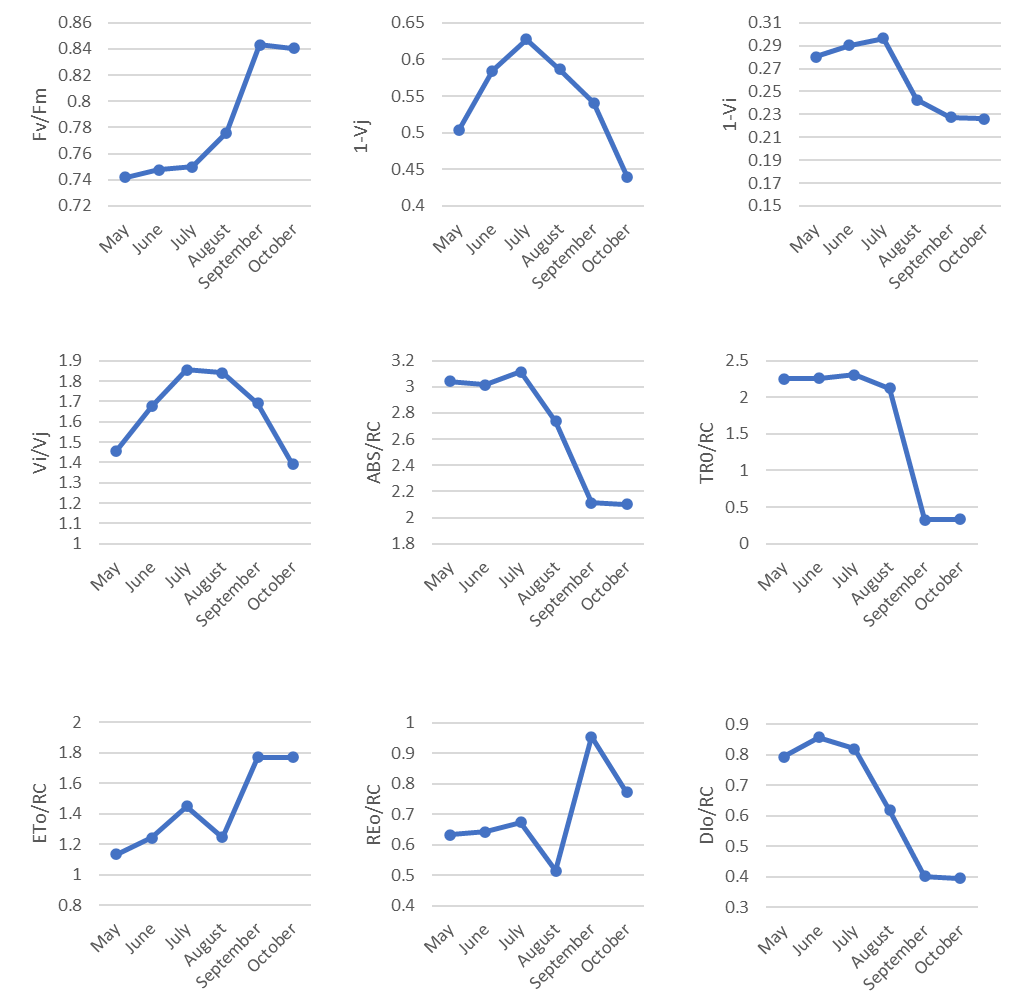


B


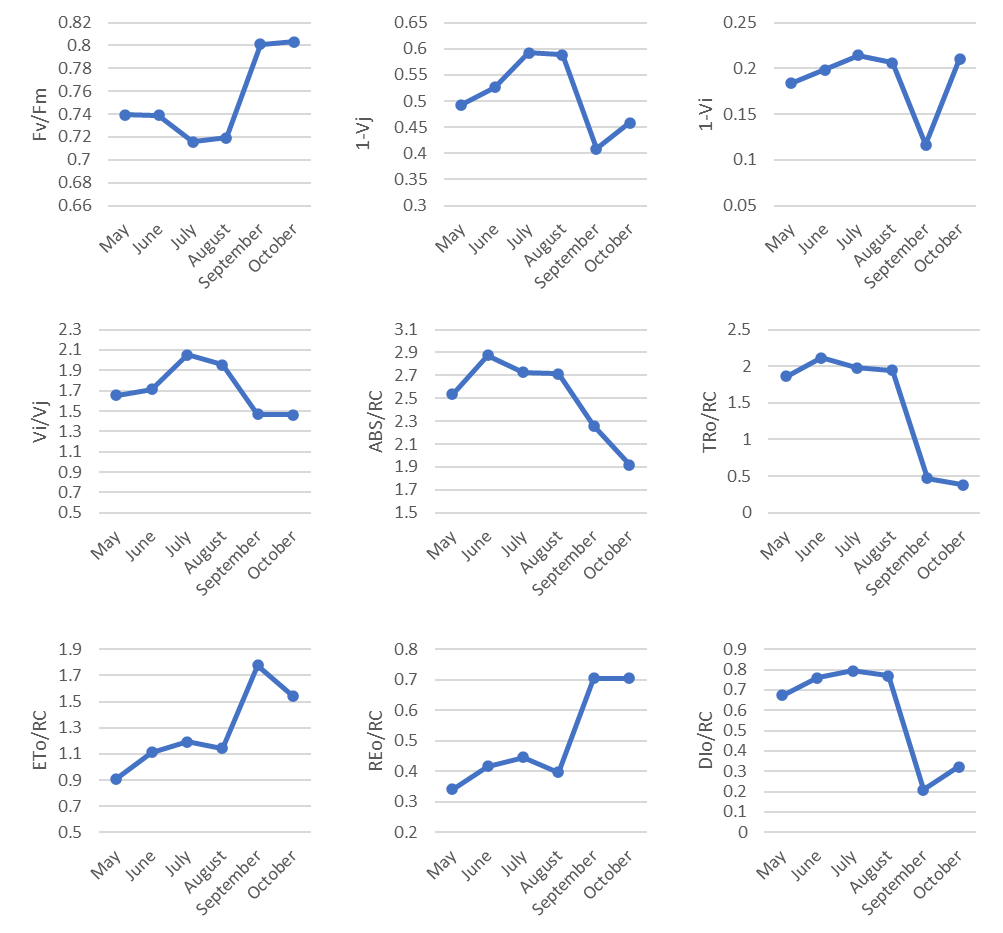


C

Table S1. Climatic data for the 2023 monitoring period (May-October) extracted from the ERA5 reanalysis dataset, and deviation values (∆) relative to the 1991-2020 baseline.

|  | **2023 Monitoring period [∆ to 1991-2020 baseline]** | | | | | | |
| --- | --- | --- | --- | --- | --- | --- | --- |
| **Parameters** | **May** | **June** | **July** | **August** | **September** | **October** | **May-October** |
| *T mean*  *(°C)* | 16.1 [+ 0.0] | 21.5 [+ 1.0] | 25.8 [+ 2.3] | 25 [+ 1.4] | 21.5 [+ 2.7] | 18.2 [+ 4.1] | 21.4 [+ 2.0] |
| *T min*  *(°C)* | 8.0 [+ 2.3] | 12.3 [+ 2.4] | 16.5 [+ 3.4] | 14.3 [+ 1.3] | 11.5 [+ 3.1] | 9.0 [+ 5.0] | 11.9 [ + 2.9] |
| *T max*  *(°C)* | 25.0 [- 1.8] | 33.4 [+ 2.2] | 35.2 [+ 1.6] | 37.6 [+ 4.2] | 31.6 [+ 2.6] | 29.3 [+ 6.0] | 32.0 [+ 2.4] |
| *Dew point T (°C)* | 12.0 [+ 1.2] | 15.5 [+ 1.5] | 17.3 [+ 2.1] | 15.7 [+ 0.1] | 14.0 [+ 1.1] | 13.6 [+3.1] | 14.7 [+ 1.6] |
| *Shortwave radiation*  *(W m^-2^)* | 180.2 [- 23.4] | 219.0 [- 8.3] | 242.8 [+ 9.2] | 206.8 [+ 3.3] | 166.2 [+ 17.1] | 104.7 [+ 9.3] | 186.6 [+ 1.2] |
| *Soil T*  *(°C)* | 13.8 [+ 0.4] | 17.9 [+ 0.8] | 22.2 [+ 1.8] | 23.2 [+ 1.3] | 21.7 [+ 2.3] | 19.2 [+ 3.2] | 19.7 [+ 1.7] |

Notes: T mean is the average air temperature at 2 meters above the surface; T min is the minimum air temperature at 2 meters above the surface; T max is the maximum air temperature at 2 meters above the surface; Dew point T is the dew point temperature at 2 meters above the surface; Shortwave radiation is the amount of solar radiation reaching the surface of the Earth (both direct and diffuse) minus the amount reflected by the Earth’s surface; Soil T is the soil temperature recorded at depths ranging from 0 to 289 cm.

Table S2. Information on clear-sky days (cloud cover < 1%) and days with maximum air temperatures exceeding 30 °C and 35 °C of the monitoring period (May-October, 2023).

|  | **2023 Monitoring period** | | | | | | |
| --- | --- | --- | --- | --- | --- | --- | --- |
| **Parameters** | **May** | **June** | **July** | **August** | **September** | **October** | **May-October** |
| *Clear-sky days*  *(n) [%]* | 9 [29] | 11 [37] | 21 [68] | 19 [61] | 16 [53] | 8 [26] | 84 [46] |
| *Days with max air T > 30 °C*  *(n) [%]* | 0 | 8 [27] | 27 [87] | 23 [74] | 10 [33] | 2 [6] | 70 [38] |
| *Days with max air T > 35°C*  *(n) [%]* | 0 | 1 [3] | 10 [32] | 14 [45] | 0 | 0 | 25 [14] |

Table S3. Results of one-way repeated measures ANOVA of Chl-a fluorescence parameters analysed.

| **Parameters** | **F** | ***P*** |
| --- | --- | --- |
| *A. donax* | | |
| REo/RC | 55.882 | < 0.001 |
| *L. nobilis* | | |
| F_V_/F_M_ | 16.753 | < 0.001 |
| 1-V_J_ | 17.403 | < 0.001 |
| 1-V_I_ | 11.819 | < 0.001 |
| ABS/RC | 18.011 | < 0.001 |
| ETo/RC | 93.272 | < 0.001 |
| REo/RC | 67.289 | < 0.001 |
| Sm | 15.472 | < 0.001 |
| *A. verlotiorum* | | |
| F_V_/F_M_ | 37.159 | < 0.001 |
| 1-V_J_ | 27.039 | < 0.001 |
| ABS/RC | 35.285 | < 0.001 |
| TRo/RC | 620.66 | < 0.001 |
| ETo/RC | 29.449 | < 0.001 |
| REo/RC | 31.652 | < 0.001 |
| PI_ABS_ | 48.404 | < 0.001 |
| Sm | 7.906 | < 0.001 |
| Fo | 15.25 | < 0.001 |

Table S4. Results of one-way repeated measures ANOVA of chlorophyll, flavonols and NBI.

| **Parameters** | **F** | ***P*** |
| --- | --- | --- |
| *A. donax* | | |
| Chlorophyll | 8.053 | < 0.001 |
| Flavonols | 27.774 | < 0.001 |
| *L. nobilis* | | |
| Chlorophyll | 23.418 | < 0.001 |
| NBI | 22.752 | < 0.001 |
| *A. verlotiorum* | | |
| Flavonols | 4.421 | 0.003 |
| NBI | 2.537 | 0.044 |

Table S5. Measured parameters from the fast-chlorophyll-*a*-fluorescence transients.

| Fluorescence parameters | Description |
| --- | --- |
| ABS/RC | The photon absorption per active reactive centre^15^ |
| TRo/RC | Electron trapping efficiency^15^ |
| ETo/RC | The electron transport activity inside of the reaction centre also^15^ |
| REo/RC | The final electron flux transferred from Q_A_^-^ to final PSI per active PSII^14^ |
| DIo/RC | The dissipation energy flux per RC^15^ |
| 1-V_J_ (ETo/TRo) | The parameter related with the electron transfer efficiency from Q_A_ to PQ when it is trapped in the PSII^14^ |
| 1-V_I_ (REo/TRo) | The electron transfer efficiency from PSII to a final acceptor PSI^14^ |
| V_I_/V_J_ (REo/ETo) | The transfer efficiency of a transferred electron to PSI from plastoquinol (PQH_2_)^14^ |
| F_V_/F_M_ | The maximum quantum yield of PSII photochemistry^15^ |
| Sm | The number of electron carriers per electron transport chain^14^ |
| PI_ABS_ | Performance index, calculated on the energy absorption basis, for the energy conservation^15^ |
| Fo | The minimal fluorescence obtained when all PSII RCs are open^30^ |

Table S6. Thermometric data used for parameter linear modeling

| **Month** | **Tmax_lag7** | **Tmean_lag7** | **Tmin_lag7** | **H_lag_o34** | **Tmax_lag0** | **Tmean_lag0** | **Tmin_lag0** |
| --- | --- | --- | --- | --- | --- | --- | --- |
| May | 22.7 | 16 | 5.9 | 0 | 17.9 | 16.7 | 13.9 |
| June | 28.02 | 19.9 | 14.1 | 0 | 24.9 | 23.2 | 15.5 |
| July | 37.2 | 27 | 19 | 19 | 33.1 | 30.9 | 22.2 |
| August | 31.8 | 23.2 | 15 | 0 | 27.6 | 24.2 | 13.4 |
| September | 30.3 | 23.6 | 17 | 0 | 22.9 | 20.9 | 17.2 |
| October | 21.3 | 12.5 | 3.9 | 0 | 12.6 | 12.5 | 11.9 |
